# Effectiveness of Cognitive Remediation in Early Versus Chronic Schizophrenia: A Preliminary Report

**DOI:** 10.3389/fpsyt.2019.00236

**Published:** 2019-04-11

**Authors:** Giacomo Deste, Stefano Barlati, Alessandro Galluzzo, Paola Corsini, Paolo Valsecchi, Cesare Turrina, Antonio Vita

**Affiliations:** ^1^Department of Mental Health and Addiction Services, ASST Spedali Civili, Brescia, Italy; ^2^Department of Clinical and Experimental Sciences, University of Brescia, Brescia, Italy

**Keywords:** schizophrenia, early course, cognitive remediation, social function, effectiveness

## Abstract

**Background:** Many evidences have demonstrated the effectiveness of cognitive remediation on cognition and functioning in patients with schizophrenia. Some researchers speculate that cognitive deficits are more amenable to remediation during earlier phases of illness than in chronicity. Therefore, cognitive rehabilitation should be used as an early intervention, seeking to produce durable functional changes in the early course of schizophrenia. Although there is strong evidence that cognitive remediation is effective in adult schizophrenia, there is little evidence about its efficacy and long-term generalized effectiveness in the early course of the disease. In this paper, we intended to investigate the possibility that cognitive remediation may produce more beneficial effects when applied in the early phase of the illness compared to chronic patients.

**Materials and methods:** Data were gathered from a database used for a previous study performed by our group, in which 56 patients with schizophrenia received a cognitive remediation intervention. In a *post hoc* analysis, patients with a duration of illness shorter than 5 years were defined as “early course” patients, while patients with a duration of illness longer than 5 years were defined as “chronic.” Clinical, neuropsychological, and functional outcome variables were assessed at baseline and after treatment.

**Result:** Of the 56 patients included in the study, 11 were “early course” and 45 were “chronic.” Both the early course group and the chronic group showed significant improvements in all the clinical, neurocognitive, and functional parameters analyzed. A significantly greater improvement in early course patients compared with chronic patients emerged in clinical and functional measures. No differential change was observed between early course patients and chronic patients in the cognitive composite score.

**Conclusion:** Our study confirms the effectiveness of cognitive remediation in improving clinical, cognitive, and functional parameters in patients with schizophrenia, both in patients in the early course and in chronic patients. However, patients in the early course showed a differential, greater change in clinical and functional parameters compared to chronic patients. Although this study has some limitations, it confirms the effectiveness of cognitive remediation interventions, particularly if applied in the early course of the illness.

## Introduction

Cognitive impairment represents a core feature of schizophrenia (1, 2), and its heavy impact on functional outcome has been widely demonstrated (3, 4). In recent years, several cognitive remediation (CR) interventions have been developed and have been used in integrated treatment approaches in patients with schizophrenia. The effectiveness of these treatments in the improvement of cognition and social functions is now well established (5, 6). However, many issues are still debated, such as the role of specific patients’ characteristics in influencing the possibility to fully benefit from the effects of cognitive rehabilitation (7–11). Among those characteristics, younger age and shorter duration of illness have been identified as predictors of the effectiveness of CR in schizophrenia. In a review by our group (12), we found preliminary positive, yet not conclusive results. In fact, although in some studies age has been found not to be related to cognitive improvement (6, 13, 14), and in others mixed results emerged (15), a number of evidences confirmed the higher possibility of younger patients to achieve cognitive improvement after CR, with patients over the age of 40 showing a poorer response to CR, compared to patients under 40 (8, 11, 16–18). Furthermore, stage of illness, a variable closely related to age, might affect cognitive improvement after CR. In a study by Corbera et al. (11), the early-stage [25 years or younger; mean duration of illness (DOI) = 3.4 years] and early-chronic [26–39 years; mean DOI = 7.6 years] patients receiving CR showed larger improvements in working memory, compared to the late-chronic group (40 years and over; mean DOI = 18.2 years). In Bowie et al. (19), early course patients (less than 5 years from the psychotic onset) showed greater improvements in processing speed and executive functions, compared to chronic patients (more than 15 years of illness) after CR. Authors concluded that duration of illness was inversely associated with improvement in cognition after a CR intervention. The aim of this paper was to compare the effects of CR interventions in patients with schizophrenia in the early course of illness and in chronic patients, with the hypothesis of greater CR benefits in patients with a shorter duration of illness.

## Methods

### Participants

Data for the present study were collected from a database originally composed for a previous study, conducted at the University Department of Mental Health of the Spedali Civili Hospital of Brescia, Italy (20), in which 84 patients with a diagnosis of schizophrenia The Diagnostic and Statistical Manual of Mental Disorders (DSM-IV) were followed naturalistically for 6 months and were randomized to a CR intervention or treatment as usual. Patients with a diagnosis of substance use disorder and mental retardation [full scale IQ lower than 70 at the Wechsler Adult Intelligence Scale-Revised (WAIS-R) (21)] or with positive symptomatology or impulsivity severity that needed hospitalization or major drug changes were excluded from the study. Patients with an age from 18 to 50 years were allowed to enter the study. Out of the 56 patients randomized to a CR intervention, 30 patients were treated with a computer-assisted CR intervention (CACR) [see Ref. (20)], and 26 received the first two sub-programs of the integrated psychological treatment (IPT). The CACR is an individualized computer-based procedure for CR, targeting cognitive functions through both domain-specific and non-domain-specific tasks. Domain-specific exercises are meant to target distinct cognitive functions among those reported to be impaired in schizophrenia patients (verbal memory, attention/vigilance, processing speed, working memory, and executive functions), while non-domain-specific tasks engage several cognitive functions at the same time. For the present study, the Cogpack software (Marker Software^®^) was used.

The IPT, on the other hand, is a manualized therapy program for schizophrenia patients, combining neuro- and social-cognitive remediation with psychosocial rehabilitation strategies; indeed, it is organized as a group approach (22). For the present study, groups of 8 to 10 patients were formed, and the cognitive subprograms of the IPT were administered each time by two trained mental health professionals.

Both IPT groups and CACR patients attended 45-min therapy sessions twice a week, for 24 weeks. For the same time, and following the same time schedule, the 28 patients randomized to treatment as usual received noncognitive specific rehabilitation, such as occupational therapy, art therapy, and physical training. However, for this study, only the 56 participants randomized to CR (i.e., the 30 patients who received CACR and the 26 who received IPT) were included in the analyses. All the patients went on receiving usual care provided by a multidisciplinary psychiatric team, including maintenance treatment with antipsychotics and rehabilitative interventions. Rehabilitation strategies (aiming at promoting the patients’ functional recovery) were individually tailored depending on clinical demands and patients’ attitudes and were delivered in a uniform way between groups (20).

Maintenance treatment was adiministered on a flexible dose schedule; the majority of patients (N = 41) received second-generation antipsychotics, while 15 patients were treated with first-generation drugs. Antipsychotics mean daily doses were reported using chlorpromazine equivalents, calculated for each patient using the method proposed by Woods (23). Use of benzodiazepines and anticholinergics was permitted when needed. Patients were assessed at study entry, and after treatments. They were assessed with measures of clinical severity, social functioning, and neuropsychological performance tests. The demographic characteristics of the sample are shown in **Table 1**.

**Table 1:** Demographic variables of the sample.

	Total	Early course	Chronic	p (t-test, chi-squared)
N	56	11	45	
M:F	40:16	9:2	31:14	0.395
IPT:CACR	26:30	5:6	21:24	0.942
Typicals:Atypicals	10:46	1:10	9:36	0.397
Chlorpromazine Equivalents	634±387	409±111	689±411	<0.001
Age	37.00±10.30	23.82±4.53	40.22±8.60	<0.001
Duration of illness (years)	14.87±9.68	2.50±1.39	17.89±8.32	<0.001
School Years	10.45±2.91	10.91±2.54	10.33±3.01	0.562
WAIS-R FSIQ	86.30±12.71	88.00±13.08	85.89±12.73	0.626

Early course, patients with a duration of illness (DOI) < 5 years; Chronic, patients with a duration of illness (DOI) > 5 years; WAIS-R FSIQ, Wechsler Adult Intelligence Scale—Revised, Full Scale Intelligence Quotient; IPT, integrated psychological therapy; CACR, computer-assisted cognitive remediation. Typicals and atypicals refer to first-generation and second-generation antipsychotics, respectively.

### Assessment

Clinical, neuropsychological, and functional assessment took place at baseline (t0) and at endpoint (6 months of follow-up), after the CR interventions.

Psychopathological assessment was performed using the Clinical Global Impression—Severity (CGI-S) scale (24) and the Positive and Negative Syndrome Scale (PANSS) (25). These scales were completed by the treating psychiatrists (not informed on which kind of CR their patients were receiving) in the psychiatric outpatients units.

As for the neurocognitive evaluation, the raters were trained professionals, external to the treatment groups and blinded to the subjects’ allocation. Before study entry, the patients were screened making use of the WAIS-R, adopted as an inclusion criterion measure (full scale IQ ≥70). Then, the included subjects underwent an exhaustive neuropsychological assessment at baseline and after 24 weeks. The following instruments were selected among those usually applied in neurocognitive evaluation of schizophrenia patients, representing a reasonable balance between comprehensiveness and ease of use (20, 26): Trail Making Test Part A (TMT-A), Trail Making Test Part B (TMT-B) (27), Wisconsin Card Sorting Test (WCST) (28), Self-Ordered Pointing Task (SOPT) (29), and California Verbal Learning Test (CVLT) (30).

Specific domains of cognitive functioning were then combined using the following four cognitive constructs: 1) processing speed: TMT-A; 2) working memory: TMT-B and SOPT, number of errors; 3) verbal memory: mean number of correct responses at immediate free recall, short- and long-delay free recall and short- and long-delay cued recall, CVLT; and 4) executive functions: TMT-B minus TMT-A (used as a flexibility index) (31), and mean percentage of perseverative and total errors, WCST. A global cognitive index was also derived by taking the average value of the other composite scores. When a neurocognitive test was not available, the relative composite score was considered as a missing value, and global cognitive score was not calculated (see the section Statistical Analysis). Z scores for each neuropsychological test were either derived using the Italian normative data for TMT and WCST (32) or control data published in previous studies for SOPT (26), or obtained in healthy subjects (N = 109) recruited by our group for CVLT.

The Z scores for each cognitive construct were calculated by taking the average of the Z scores of the specific corresponding tests (see 20). Finally, psychosocial functioning outcome measures were assessed by the referring multidisciplinary rehabilitative team, who usually took care of the patients and provided their standard rehabilitative interventions in the outpatient settings. This team did not include any personnel involved in the administration of the experimental CR programs and was also blinded to the patients’ allocation. Evaluations were completed with team consensus, and every professional involved in the study was trained in the use of the rating instruments. The functional outcome measures used were the Global Assessment of Functioning (GAF) scale (33) and the Health of the Nation Outcome Scale (HoNOS) (34, 35).

### Statistical Analysis

The analyses were performed only in the group of patients who received a CR intervention (N = 56). To test the hypothesis that patients in the early course of the illness could take more advantage from CR compared to chronic patients, participants were divided into two groups, based on the duration of illness. Patients with a duration of illness shorter than 5 years were defined as “early course,” while patients with a duration of illness longer than 5 years were defined as “chronic.”

This cutoff of 5 years was chosen according to literature on early course definition in schizophrenia and CR in early course patients with schizophrenia (19, 36).

Duration of illness was calculated starting from the first psychotic episode. Data regarding duration of illness were acquired by patients themselves, relatives, medical records, and health care professionals, involved in the routine care of the patients.

Demographic variables at baseline were compared between groups (early course and chronic) using t tests and chi-squared tests as appropriate. Clinical, neurocognitive, and psychosocial functioning variables at baseline were also compared between groups using t tests.

Within-group changes of clinical, neurocognitive, and functional variables were analyzed using pared samples t tests. Clinical, neurocognitive, and functional changes were compared between the two groups using repeated-measures analysis of variance, covaried by baseline. p values < 0.05 (two-tailed) were considered significant. Statistical analyses were conducted using SPSS 14.0 software.

## Results

Of the 56 patients included in the study, 11 were in their first 5 years of illness and thus were defined as “early course,” while the other 45 were defined as “chronic,” having a duration of illness longer than 5 years. Early course patients had a lower mean age, had a shorter mean duration of illness, and received a lower antipsychotic (chlorpromazine equivalents) mean daily dose. No differences between intervention (IPT and CACR) distribution, type of antipsychotics distribution (first- and second-generation antipsychotics), sex distribution, mean school years, and WAIS-R FSIQ emerged between early course and chronic patients (**Table 1**). A higher score at the PANSS negative and general psychopathology subscales and at the PANSS total score emerged in the early course group compared to chronic patients (**Table 2**). No baseline differences in any other clinical (CGI-S, PANSS positive subscale), neurocognitive, and psychosocial functioning variables emerged between groups. Significant (p < 0.05) within-groups improvements in all the clinical, neurocognitive, and functional parameters analyzed using the paired samples t tests emerged in both the early course group and the chronic group. A significantly greater improvement in early course patients compared with chronic patients emerged for CGI-S, PANSS total score, PANSS positive subscale, PANSS negative subscale, PANSS general psychopathology subscale, GAF, and HoNOS total score. No differential change was observed between early course patients and chronic patients in the Global Cognitive Composite Score.

**Table 2:** Between-group comparisons of change of clinical, neurocognitive, and psychosocial functioning variables.

	T0	T1	Repeated-measures ANCOVA (covaried by baseline) time × group interaction p	Effect size (partial eta squared)
CGI-S (early course)	5.09 ± 0.53	3.50 ± 0.70	0.002	0.172
CGI-S (chronic)	4.76 ± 0.74	3.98 ± 0.83		
PANSS Pos (early course)	19.45 ± 5.42	11.90 ± 3.34	0.028	0.092
PANSS Pos (chronic)	18.87 ± 5.25	13.65 ± 3.37		
PANSS Neg (early course)	31.36 ± 6.80*	18.60 ± 8.05	0.007	0.137
PANSS Neg (chronic)	23.78 ± 7.61	18.23 ± 4.83		
PANSS Gen (early course)	51.45 ± 10.44*	31.90 ± 8.81	<0.001	0.225
PANSS Gen (chronic)	44.20 ± 8.44	35.07 ± 7.73		
PANSS Tot (early course)	102.27 ± 17.90*	62.40 ± 17.99	0.001	0.190
PANSS Tot (chronic)	86.84 ± 15.63	66.95 ± 12.29		
GAF (early course)	41.09 ± 9.42	56.10 ± 8.43	0.006	0.138
GAF (chronic)	47.91 ± 10.53	55.14 ± 8.92		
HoNOS (early course)	19.55 ± 4.92	7.80 ± 5.63	0.010	0.127
HoNOS (chronic)	17.80 ± 5.39	10.91 ± 5.96		
Global cognition (early course)	−0.70 ± 0.79	−0.32 ± 0.92	0.648	0.004
Global cognition (chronic)	−1.21 ± 0.93	−0.63 ± 0.93		
Processing speed (early course)	0.10 ± 0.47	0.29 ± 0.66	0.871	0.001
Processing speed (chronic)	−0.36 ± 1.27	0.02 ± 0.80		
Working memory (early course)	−0.54 ± 1.11	−0.39 ± 1.16	0.693	0.003
Working memory (chronic)	−1.24 ± 1.05	−0.69 ± 1.07		
Verbal memory (early course)	−1.96 ± 1.31	−1.17 ± 1.68	0.570	0.006
Verbal memory (chronic)	−2.58 ± 1.46	−1.43 ± 1.68		
Executive functions (early course)	−0.40 ± 0.84	−0.02 ± 0.69	0.134	0.045
Executive functions (chronic)	−0.67 ± 0.94	−0.45 ± 0.95		

Early course, patients with a duration of illness (DOI) < 5 years; Chronic, patients with a duration of illness (DOI) > 5 years.

*Baseline between groups difference, t test, p < 0.05.

## Discussion

This study confirms the effectiveness of CR in improving clinical, cognitive, and functional parameters in patients with schizophrenia. This effectiveness is demonstrated in patients in the early course of the illness as well as in chronic patients. However, patients in the early course showed a differential, greater change in clinical and functional parameters compared to chronic patients. In fact, it is possible that the group in the early course of the illness may benefit from the advantage of a younger age, with this parameter being a well-known predictor of functional improvement after CR (8). However, although both early course and chronic patients improved in global cognitive performance, no between-groups differences emerged in the change of such parameter. Even if this result confirms the possibility for patients with schizophrenia to benefit from CR both in the early phase of the illness and in later stages, it is not in line with previous evidences, reporting greater cognitive improvements in patients with a shorter duration of illness compared to chronic patients after CR (11, 19) and, more in general, with studies that suggest that psychosocial improvements after CR may be mediated by cognitive improvements (20).

Furthermore, the baseline greater severity of negative and general psychopathology observed in the early course group compared to chronic patients, a factor found to be associated to less marked cognitive improvements after CR (18), could have represented a potentially limiting factor in detecting between-groups differences in cognitive change after CR. Conversely, the lower antipsychotic mean daily dose that emerged in the early course group, a factor that has been found to be associated to greater cognitive and psychosocial functioning amelioration after CR (8), in this case should not be considered as an indirect proxy of symptoms severity and suggests a more specific role of the antipsychotic treatments in psychosocial functioning improvement after CR, a hypothesis that should be better analyzed in future studies.

This study has several limitations: first, the small sample size could have limited the statistical power of the analyses and the possibility to perform further, potentially interesting analyses, such as the comparison between type of intervention (IPT and CACR) in early course and chronic patients; second, the possibility to generalize the results may be restricted by the specific sample recruited for the study, including patients followed in the Italian psychiatric rehabilitation services; third, the original study was not explicitly designed with the purpose of comparing the differences of the effects of CR between patients with schizophrenia in their early course of illness and chronic patients; fourth, being an exploratory study, a correction for multiple comparisons was not used, in order to avoid the possibility of missing potentially interesting results, to be further analyzed in future studies; fifth, the cutoff for early course patients, although not one of the strictest among those proposed in literature (36), did not allow the identification of two groups of identical size, thus further limiting the statistical approach. Nevertheless, in a recent review about the diverse definition of the early course of schizophrenia, the authors suggested that disease duration of <5 years encompasses the previous definition of the critical period for early intervention (36).

Despite these limitations, the results of the study clearly suggest that benefits from CR may be better when these interventions are applied in patients with schizophrenia at their early stages of the illness. These results, if confirmed by further studies, specifically designed for this purpose, point towards the perspective of earlier interventions in psychosis, with the possibility to also use non-pharmacologic evidence-based treatments that may also be potentially useful not only in the early course of schizophrenia but also in patients defined at risk of psychosis (12).

## Ethics Statement

Written informed consent to treatment was obtained from all participants after the nature of the intervention procedures had been fully explained. The project was approved by the Board for Innovation in Psychiatry of the Health Authority of the Lombardia Region, Italy. The work has been carried out in accordance with the Code of Ethics of the World Medical Association.

## Author Contributions

AV designed the project, and reviewed and discussed the data and statistical analyses and the final version of the paper. GD administered and scored neuropsychological tests, prepared the database, participated in the analyses, and wrote the paper. SB and PC followed patients in the rehabilitative interventions. AG, PV, and CT participated in the discussion of the data and manuscript. All authors contributed to and approved the final manuscript.

## Funding

Funding for this study was partially provided by an unrestricted grant from the Lombardia Region (project TR11 and project 195) and by a 60% grant from the University of Brescia (School of Medicine).

## Conflict of Interest Statement

The authors declare that the research was conducted in the absence of any commercial or financial relationships that could be construed as a potential conflict of interest.
